# Age-Related Changes in Perirhinal Cortex Sensitivity to Configuration and Part Familiarity and Connectivity to Visual Cortex

**DOI:** 10.3389/fnagi.2017.00291

**Published:** 2017-09-15

**Authors:** Laura Cacciamani, Erica Wager, Mary A. Peterson, Paige E. Scalf

**Affiliations:** ^1^Department of Psychology and Child Development, California Polytechnic State University San Luis Obispo, CA, United States; ^2^Department of Psychology, University of Arizona Tucson, AZ, United States; ^3^Cognitive Science Program, University of Arizona Tucson, AZ, United States; ^4^Department of Psychology, Durham University Durham, United Kingdom

**Keywords:** perirhinal cortex, object perception, familiarity, functional connectivity, visual cortex, parts and wholes

## Abstract

The perirhinal cortex (PRC) is a medial temporal lobe (MTL) structure known to be involved in assessing whether an object is familiar (i.e., meaningful) or novel. Recent evidence shows that the PRC is sensitive to the familiarity of both whole object configurations and their parts, and suggests the PRC may modulate part familiarity responses in V2. Here, using functional magnetic resonance imaging (fMRI), we investigated age-related decline in the PRC’s sensitivity to part/configuration familiarity and assessed its functional connectivity to visual cortex in young and older adults. Participants categorized peripherally presented silhouettes as familiar (“real-world”) or novel. Part/configuration familiarity was manipulated via three silhouette configurations: *Familiar* (parts/configurations familiar), *Control Novel* (parts/configurations novel), and *Part-Rearranged Novel* (parts familiar, configurations novel). “Real-world” judgments were less accurate than “novel” judgments, although accuracy did not differ between age groups. The fMRI data revealed differential neural activity, however: In young adults, a linear pattern of activation was observed in left hemisphere (LH) PRC, with *Familiar* > *Control Novel* > *Part-Rearranged Novel*. Older adults did not show this pattern, indicating age-related decline in the PRC’s sensitivity to part/configuration familiarity. A functional connectivity analysis revealed a significant coupling between the PRC and V2 in the LH in young adults only. Older adults showed a linear pattern of activation in the temporopolar cortex (TPC), but no evidence of TPC-V2 connectivity. This is the first study to demonstrate age-related decline in the PRC’s representations of part/configuration familiarity and its covariance with visual cortex.

## Introduction

The ability to discriminate an object that is familiar and meaningful from one that is novel and meaningless (for instance, for objects that are newly encountered) is crucial to our interaction with the world. The existence of and access to stored information from previous encounters with an object can modulate the perceptual processing of that object and the generation of appropriate responses to it (e.g., Peterson, 1994).

Previous studies have shown that normal aging compromises this ability to create, access and use information about objects, especially when they share features or parts with other objects. Compared to their younger counterparts, older adults have difficulty learning to distinguish two objects that have multiple features in common—or are high in “feature ambiguity”—and therefore must be discriminated based on the configuration of those features (Ryan et al., 2012; Scheerer and Marrone, 2014; see also Insel et al., 2008). This finding reflects an age-related deficit in creating new object representations under complex, ambiguous conditions. Likewise, when monkeys are shown two similar LEGO^®^ objects and must learn to associate one object with a food reward, age-related deficits arise when two objects have more than 85% of features in common (Burke et al., 2011). Although older monkeys are able to remember the objects once learned, they require more trials to learn to discriminate the rewarded from the unrewarded object. This likely reflects their difficulty in perceiving minor differences between objects with high feature ambiguity. Other work in humans has shown that older adults have difficulty maintaining newly formed object representations after a delay (Soldan et al., 2009; Gordon et al., 2013), suggesting that the age-related decline in object processing transcends both perception and memory.

Constructing and utilizing representations of both previously encountered and currently present objects is known to be critically dependent on the perirhinal cortex (PRC) of the medial temporal lobe (MTL). Neurophysiological research has demonstrated that monkeys and rats with selective lesions to the PRC are especially impaired on visual object discrimination tasks when feature ambiguity is high (Bussey and Saksida, 2002; Bussey et al., 2002; Bartko et al., 2007; Murray et al., 2007). Similarly, humans with damage to the MTL that includes the PRC exhibit impairments on odd-one-out discrimination tasks, particularly when the objects have multiple features in common, whereas humans with damage restricted to the hippocampus do not exhibit this deficit (Lee et al., 2005; Barense et al., 2007; see also Kivisaari et al., 2012). Functional magnetic resonance imaging (fMRI) studies have further shown that blood oxygen level dependent (BOLD) activity in the PRC is higher during an object discrimination task if the objects have high vs. low feature ambiguity, even if level of difficulty is controlled (Barense et al., 2010).

Prior work has shown that the functioning of the PRC declines with age, and this deterioration may partially explain age-related object perception deficits. In rats, cellular function in the PRC seems to weaken with age (Liu et al., 2009; Rushaidhi et al., 2012). Additionally, neuroimaging studies in humans have demonstrated that during an object discrimination task under conditions of high feature ambiguity (i.e., when age-related deficits arise), engagement of the PRC is substantially reduced in older vs. younger participants (Ryan et al., 2012).

**Figure 1:**
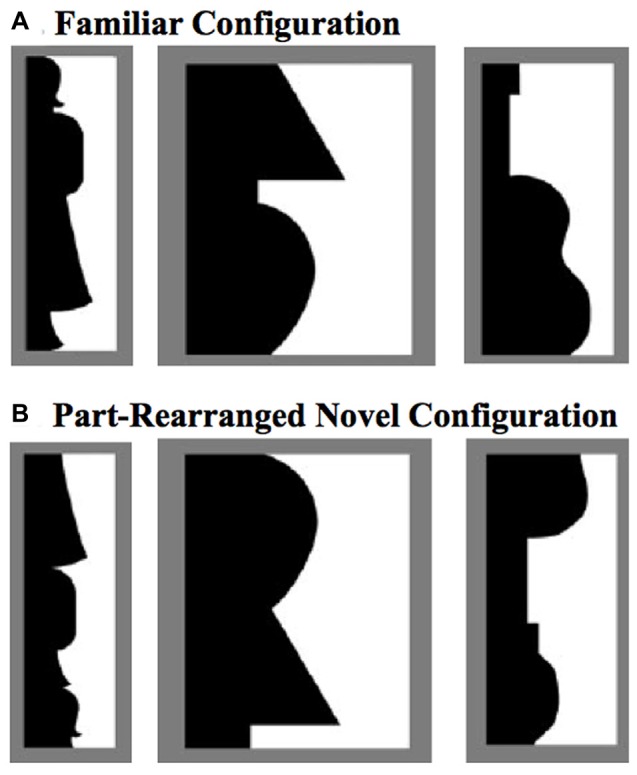
Example stimuli used to test effects of familiarity on object perception (Peterson and Gibson, 1994; Peterson et al., 1998, 2000). **(A)** *Familiar Configurations* (composed of familiar parts), with the critical region (black here) depicting (from left to right) a standing woman, a lamp, and a guitar. **(B)** The black regions depict *Part-Rearranged Novel Configurations*, composed of the same familiar parts as the objects in **(A)**, but here they are spatially rearranged so that the configuration is novel. The critical regions here are shown in black and on the left, but color and side were counterbalanced in the experiment.

The PRC is involved not only in discriminating between two similar objects, but also in using existing representations of familiar objects to aid in segregating those objects from their backgrounds. For instance, when presented with displays like those in Figure 1 and asked whether the central border shapes an object on the left or right side, non brain-damaged participants are more likely to perceive the object on a given side if a *Familiar Configuration* is depicted there (Figure 1A) than if the same familiar parts are rearranged there to form a novel configuration (*Part-Rearranged Novel Configurations*; see Figure 1B; Gibson and Peterson, 1994; Peterson et al., 1998, 2000). The difference in perception between displays containing *Familiar* and *Part-Rearranged Novel Configurations*—an effect we refer to as a *configural familiarity* effect—is significantly reduced in patients with MTL damage that includes the PRC, but not in patients with MTL damage restricted to the hippocampus (Barense et al., 2012). This effect arose primarily due to the PRC-damaged patients’ increased reports of *Part-Rearranged Novel Configurations* as objects, indicating that damage to the PRC caused patients to implicitly use part familiarity rather than configural familiarity for object segregation. To account for this finding, Barense et al. (2012) proposed a model in which the intact PRC modulates part familiarity responses at lower levels, reducing lower-level part familiarity responses if the configuration is novel and enhancing lower-level part familiarity responses if the configuration is familiar. When the PRC is damaged, lower-level familiarity responses for the parts of part-rearranged configurations remain high and capable of influencing object perception.

**Figure 2:**
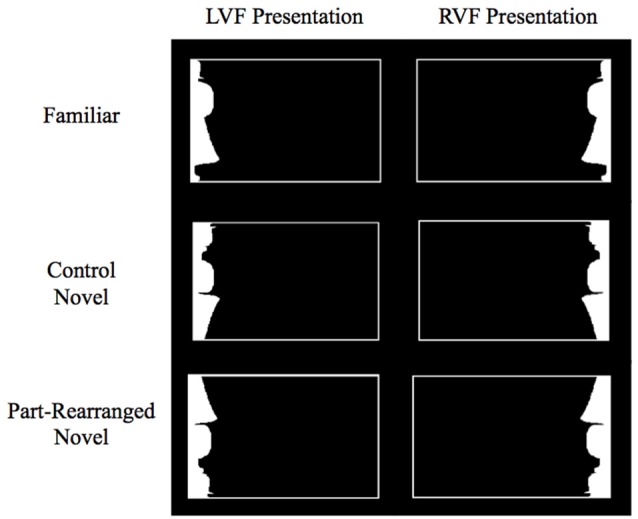
Example stimuli in each condition in Peterson et al. (2012) and the current study. Subjects were asked to fixate on a cross in the center of the screen during stimulus presentation. Stimuli were lateralized to the left visual field (LVF) or right visual field (RVF). Exemplar shown here is the standing woman.

Taken alone, Barense et al. (2012) findings did not require an explanation in terms of feedback processing; they were consistent with the feedforward models of Bussey and Saksida (2002) (Cowell et al., 2006) as well. Using fMRI, Peterson et al. (2012) found evidence consistent with Barense et al. (2012) proposal that the PRC modulates lower-level part familiarity responses. Participants viewed displays like those depicted in Figure 2. These displays were designed such that the white regions would necessarily be perceived as silhouette objects (they were smaller than the surrounding region and high contrast against the background). Participants fixated a central cross and judged whether the white silhouettes depicted portions of nameable objects that exist in the real-world or novel objects. Three types of stimuli were used in order to manipulate configural and part familiarity: *Familiar Configurations*, in which both the parts and the configuration were familiar; *Control Novel Configurations*, in which both the parts and the configuration were novel; and *Part-Rearranged Novel Configurations*, in which the parts were familiar but the configuration was novel. (Peterson et al., 2012) found that when the stimuli were in the right visual field (RVF), activation in bilateral PRC was greatest for *Familiar Configurations*, at baseline for *Control Novel Configurations*, and below baseline for *Part-Rearranged Novel Configurations*. The stimuli in the latter two conditions are both novel at the configuration level; had the PRC only been responsive to configural familiarity/novelty, activation should not have differed. The observed linear pattern of activation indicates that the PRC is sensitive to not only the familiarity/novelty of the whole configuration but also of the parts that constitute that configuration, which is not predicted by feedforward models. Further consistent with the Barense et al. (2012) hypothesis involving feedback, this pattern of activation—specifically, higher activation for the same parts present in *Familiar* than *Part-Rearranged Novel* *Configurations*—was also observed in left hemisphere (LH) V2, where receptive fields (RFs) are only large enough to encompass approximately one part (~2°) of the silhouette at the eccentricity used (3.5°). Because the *Familiar* and *Part-Rearranged Novel* *Configurations* comprise the same parts and only differ at the level of configuration, Peterson et al. (2012) suggested that the differences in activation in V2 may be due to feedback from higher levels where the configuration is represented. The PRC was a strong candidate for this role, given that its activation pattern so closely resembled that observed in V2. In support of this PRC-V2 feedback hypothesis, one recent study did find evidence of directed functional connectivity between the PRC and the visual cortex (Cacciamani and Likova, 2017); however these results were observed in blind individuals and only after extensive cognitive training. Evidence for PRC-V2 functional connections in normally sighted young adults at baseline—as well as the effect of aging on these connections—has yet to be found.

In the current experiment, we investigate whether aging compromises PRC and V2 sensitivity to object familiarity. We tested older and younger participants on the real-world/novel object task and stimuli used by Peterson et al. (2012) see Figure 2) in order to assess the age-dependent stability of the PRC’s sensitivity to both configural and part familiarity and its ability to modulate lower-level responses. Based on previous work showing that aging compromises PRC functioning (Liu et al., 2009; Rushaidhi et al., 2012; Ryan et al., 2012), we expected the sensitivity of the PRC to the joint familiarity of the configurations and parts used by Peterson et al. (2012) to be reduced in older adults. Structurally, the PRC itself appears to remain stable with age (Insausti et al., 1998; Rapp et al., 2002); however, its anatomical connections (in addition to its functioning) may undergo age-related deterioration. Diffusion tensor imaging, for instance, reveals age-related decline in the integrity of the inferior longitudinal fasciculus—the white matter tracts connecting the occipital lobe to the temporal lobe, including the MTL (Voineskos et al., 2012). These studies provide insight into the age-related decline in functioning of the PRC and its structural connections. Nevertheless, no research has yet investigated age-related decrements in *functional* connectivity between the PRC and the visual cortex. In the current experiment, we examine for the first time both whether the LH PRC and V2 are functionally connected in young adults and whether this connectivity is reduced in older adults. We examine functional connectivity in the LH, where Peterson et al. (2012) previously found evidence of top-down modulation.

In addition to the PRC, we assessed age-related changes in activation in another MTL structure—the temporopolar cortex (TPC; Brodmann area 38). Our previous work showed that the TPC was also sensitive to the familiarity/novelty of both the configuration and its parts. Specifically, with left visual field (LVF) presentation, the TPC differentially activated to the different conditions, whereas the PRC did not. The pattern of activation in bilateral TPC was the opposite of that observed in bilateral PRC for RVF presentation, with highest activation for *Part-Rearranged Novel*, lower activation for *Control Novel* and lowest activation for *Familiar Configurations*. This is not the first time the two hemispheres have been shown to respond in opposite directions to familiar and novel objects (see Dien, 2008). Furthermore, with LVF presentation, activation in right hemisphere (RH) V2 mimicked the pattern observed in the TPC, which again suggests top-down modulation of lower-level familiarity responses, this time in the direction mirroring RH TPC activity. Accordingly, we included the TPC in the analysis in the present study in order to assess whether it undergoes the same age-related decline in sensitivity to configural/part familiarity as the PRC.

Previous research has shown that the TPC indeed plays a role in object familiarity. For instance, neurons in the TPC are selectively responsive to complex visual stimuli (Nakamura et al., 2000). In monkeys, the cooling of the TPC—which effectively reduces the ability of the neurons to fire—impairs monkeys’ ability to learn to discriminate novel objects, but not their ability to discriminate previously learned objects (Horel et al., 1984). Neuroimaging studies with humans have shown that the TPC is engaged during the discrimination of unfamiliar and familiar faces and scenes (Nakamura et al., 1994). Moreover, degeneration of the TPC often results in semantic dementia—a condition characterized by a loss of conceptual knowledge about real-world items (for a review, see Hodges and Patterson, 2007). Together, these studies point towards the TPC’s involvement in processing familiar objects. Additionally, like the PRC, the TPC has been shown to undergo age-related decline. Its structural integrity and resting metabolic rate are reduced with age (Eberling et al., 1995; Insausti et al., 1998; Allen et al., 2005; Fjell et al., 2009), and lower perfusion rates in the TPC have been correlated with impaired visuospatial processing (Alegret et al., 2010). Here, we further assessed whether normal aging compromises the contributions of the TPC to visual perception—specifically, the sensitivity to configural as well as part familiarity.

## Materials and Methods

### Participants

The volunteers recruited for this study were 12 young (8 females, ages 19–30) and 12 older (9 females, ages 60–85) adults. All participants were right-handed and had normal or corrected-to-normal vision. Before participating, volunteers gave written informed consent, which was approved by the Institutional Review Board of the University of Arizona. Five participants (1 young, 4 old) were eliminated due to having fewer than five correct trials in each condition^1^. Thus, data from 11 young (7 females; ages 19–30, median age = 24) and eight older (5 females; ages 60–77, median age = 68.5) participants were included in the analyses described below. The number of participants included is consistent with the number typically used for fMRI experiments that investigate targeted hypotheses in specific brain regions (e.g., Baldassano et al., 2016); in this experiment, we test two specific hypotheses involving three regions of interest (ROIs; V2, PRC and TPC) that are based on previous research (Peterson et al., 2012).

### Stimuli

The stimuli were those used by Peterson et al. (2012) and consisted of white silhouettes presented on the right or left side inside a thin white rectangular border (Figure 2). The screen background was black throughout the experiment. A white fixation cross was displayed in the center of the screen during stimulus presentation. The white silhouette and its border were shifted up 3° from this central fixation in order to target the ventral visual stream. Silhouettes subtended 6° in height and an average of 2.95° in width, and the center-most edge of each silhouette was always located 3.5° from center. Using these elongated, lateralized displays promoted the perception of the white silhouette as figure and increased the precision of the retinotopic assignment of its visual representations.

Three types of configurations were depicted by the white silhouettes: *Familiar Configurations, Part-Rearranged Novel Configurations* and *Control Novel Configurations* (see Figure 2). The *Familiar Configurations* portrayed portions of objects whose whole configurations and their parts were likely to be familiar to participants (i.e., a standing woman, lamp, guitar; see Peterson et al., 2000). The *Part-Rearranged Novel Configurations* were created by dividing the *Familiar Configurations* into parts at minima of curvature and spatially rearranging the familiar parts to form a novel configuration. The *Control Novel Configurations* were created by inverting the *Part-Rearranged Novel Configurations*; consequently, both the parts and the whole configuration were novel^2^. There were 24 unique stimuli presented in each of the three configurations, and each stimulus was presented once in the RVF and once in the LVF. There were therefore six stimulus categories total, with 24 stimuli in each category: LVF *Familiar*, RVF *Familiar*, LVF *Part-Rearranged Novel*, RVF *Part-Rearranged Novel*, LVF *Control Novel*, RVF *Control Novel Configurations*.

### Experimental Design and Equipment

**Figure 3:**
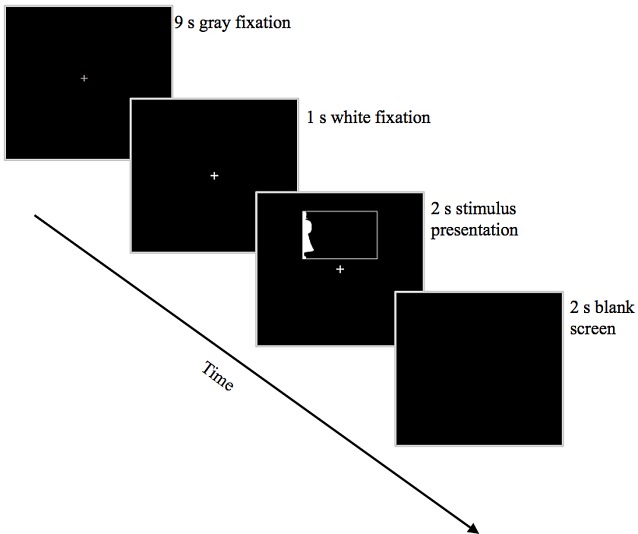
Trial structure. Depicted here is an example trial showing a *Familiar Configuration* in the LVF.

Given our small number of stimuli, we employed a slow event-related trial design (see Figure 3). Each trial began with a 10-s fixation period; 1 s before stimulus onset, the fixation cross changed from gray to white to alert the participant to the upcoming stimulus. Participants were instructed to maintain fixation on this white cross throughout the 2-s stimulus presentation, after which the screen went black for 2 s. Participants indicated whether the silhouette depicted a real-world object or a novel object using a button-box held in their right hand. One button was assigned to “yes/real-world object” and the other was assigned to “no/novel object.” Participants had 4 s from stimulus onset to respond before the next fixation cross appeared; failure to respond within this time was considered an error. Button responses were collected using a Lumina (Cedrus Corp) response pad and controller. Participants viewed the stimuli on a ThinkVision 1920 × 1200 LED monitor via rear projection onto a mirror positioned above the head coil. Stimuli were presented using Experiment Builder software running under Windows 7 Professional. To ensure that participants were maintaining fixation and not looking toward the lateralized silhouettes, eye movements were monitored in the scanner for 10 of the participants (approximately half in each age group: 6 young, 4 older) using Eyelink 1000 eye-tracking software via a long-range mount positioned at the back of the magnet. Eye movements for the remaining nine participants could not be consistently tracked due to difficulties in reliably isolating the pupil, usually because the head coil cast a shadow over the eye, reducing illumination from the infrared camera.

Participants performed six runs of 24 trials each. In each run were eight trials of each configuration type (*Familiar*, *Part-Rearranged Novel* and *Control Novel Configurations*), with half presented in the RVF and half presented in the LVF. A Latin square design was used to assign individual stimuli to each run; one and only one version of the six possible stimulus categories (i.e., LVF *Familiar*, RVF *Familiar*, LVF *Part-Rearranged Novel*, RVF *Part-Rearranged Novel*, LVF *Control Novel*, RVF *Control Novel Configurations*) of each stimulus appeared in each of the six runs (e.g., the standing woman appeared in only one of those six versions in a given run). Stimuli were randomized within each run.

### Practice and Eye-Fixation Training

Twenty-four to forty-eight hours before the scanning session, participants underwent a training session outside the scanner during which they viewed four examples each of “real-world” and “novel” silhouettes. They were told that real-world silhouettes clearly portrayed namable, common objects, while novel silhouettes were created in the lab and would have little-to-no resemblance to common namable objects. Participants were instructed to categorize subsequent silhouettes on this basis.

After these examples and instructions, participants were given 24 practice trials using different stimuli from those used during the training session outside the scanner and also different from those that would be used during the experimental scanning session. Feedback was provided on their performance. Critically, eye movements were monitored during these practice trials using a desktop-mounted Eyelink 1000 eye-tracker; if the participant’s gaze deviated from fixation during the two-second stimulus presentation, an error tone was played over their headphones, and the trial started over. This quickly trained participants to maintain fixation and resist the urge to look toward the peripheral stimulus. Training was completed only when the participant had performed all 24 trials while maintaining fixation. Eye-fixation training was conducted using Experiment Builder software running on a Pentium 4 Dell personal computer. Participants were reminded of this eye-fixation training session immediately prior to the experimental scanning session.

### Data Acquisition and Analysis

Images were collected on a 3.0 T Siemens Skyra whole-body scanner using a 32-channel head coil. An echo planar imaging (EPI) pulse sequence (TR = 3000, TE = 27, FOV = 240 × 240 mm, 1.8 mm with a 0.9 mm skip) was used to acquire 27 axial-oblique slices. Each of the six scanning runs contained 124 repetitions. To assist in image registration, a T2 image coplanar with the functional slices was also collected.

FMRIB (Oxford University Centre for Functional MRI of the Brain) Software Library (FSL) was used to analyze the functional data. Data were brain-extracted, high-pass filtered (sigma = 60 s; cut-off frequency = 100 s), intensity normalized, and motion corrected using MCFLIRT [FSL 4.1.9 (Jenkinson et al., 2002; Smith et al., 2004)]. Data were concatenated across all six runs and submitted to a GLM analysis using FSL’s FMRI Expert Analysis Tool (FEAT) v.5.98 (FSL 4.1.9; Woolrich et al., 2000; Smith et al., 2004). We modeled six regressors of interest corresponding to our six stimulus categories (RVF *Familiar*, RVF *Part-Rearranged Novel*, RVF *Control Novel*, LVF *Familiar*, LVF *Part-Rearranged Novel*, LVF *Control Novel Configurations*). These regressors were convolved with a double-gamma model of the HRF (Phase 0 s). In order to equate the number of trials in each condition for each visual field, we removed not only trials that were incorrectly categorized in one of its permutations (e.g., a “novel” response to an RVF *Familiar Configuration*), but also their counterparts in their other permutations (e.g., the corresponding RVF *Part-Rearranged Novel* and RVF *Control Novel Configurations*) and added them to a regressor of no interest. This regressor was designed to simultaneously equate statistical power across conditions of interest as well as to remove contaminating error trials from the error term of our GLM; the regressor in and of itself has no theoretical meaning. Condition-specific error trials were of insufficient power (less than eight per cell) to merit individual modeling. The resulting statistical maps were registered into the participant’s individual anatomical space and into standard space using *FMRIB’s Linear Image Registration Tool* (FLIRT; Jenkinson et al., 2002).

#### Medial Temporal Lobe Analysis Procedures

Based on the visual field effects (and resulting lateralized activation) found using this paradigm in Peterson et al. (2012), we analyzed the BOLD data from RVF and LVF presentations separately in a lower-level analysis in each participant, with a regressor for each condition (*Familiar*, *Control Novel, Part-Rearranged Novel)*. The resulting statistical maps for RVF and LVF stimulation for all participants were fed into separate mixed-design higher-level ordinary least squares (OLS) group analyses by FMRIB’s Local Analysis of Mixed Effects (FLAME). As in Peterson et al. (2012), our regressor of interest was a linear model (*Familiar*, *Control Novel, Part-Rearranged Novel*; vector 1, 0, −1). A linear model was used in order to locate areas sensitive to the conjunctive status of configural familiarity and part familiarity, rather than simply overall familiarity or novelty. Consequently, *Control Novel Configuration* served as baseline against which to judge the relative activation of *Familiar* and *Part-Rearranged Novel Configurations*. We also modeled out each participant’s mean activation level as a regressor of no interest. Data from each visual field were modeled separately. This higher-level group analysis produced statistical maps for the linear model within each age group (old and young) as well as for the contrasts between age groups (old > young; young > old).

Based on our previous research on the MTL (Peterson et al., 2012), we identified two ROIs in which we searched for a significant linear trend: the PRC and the TPC. The PRC ROI was based on the PRC probability map created by Devlin and Price (2007) and used by other researchers employing fMRI to investigate PRC function (e.g., Barense et al., 2011; see Supplementary Figure S1A). The TPC ROI was defined using BA 38 (in each hemisphere) from the Talairach-to-MNI conversion digital atlas (Lancaster et al., 2000), which was projected into each participant’s functional space, producing a larger probabilistic map that was then transformed into standard MNI space. These transformations were then averaged across all participants to produce ROIs that directly reflected the individual anatomical spaces of our participant population (see Supplementary Figure S1B).

After anatomically defining our PRC and TPC ROIs in each hemisphere, we used easythresh (FEAT 5.98) to identify clusters of voxels in these ROIs that showed a linear trend in activation (*Z* > 1.96) whose extent was greater than would be predicted by random variation in activation (*p* < 0.05), as well as clusters showing a significant difference in the linear trend between young and older participants (i.e., the young vs. old contrasts). The cluster-based analysis method (FLAME OLS) has been suggested to be vulnerable to type I error (Eklund et al., 2016) when applied in the absence of an *a priori* prediction about where activation should occur. Our experiment is designed to replicate our previous findings in young adults (Peterson et al., 2012), however. Any finding of the predicted replication is therefore unlikely to reflect type I error and meets recent demands for reproducibility in psychological science (Open Science Collaboration, 2015).

#### Visual Cortex Analysis Procedures

Each volunteer also participated in a phase-encoded retinotopic mapping session. The procedure for this scan was derived from Sereno et al. (1995). Briefly, participants viewed a flickering black-and-white checkerboard wedge extending outwards from a central fixation cross to the edge of the screen. The wedge rotated clockwise at a rate of one rotation per minute, stimulating early visual areas in a predictable pattern. Participants were asked to fixate on the central cross and attend to the rotating wedge. During this session, an EPI pulse sequence (TR = 3.05, TE = 30, FOV = 240 × 240 mm) was used to acquire one run of 30 coronal slices starting from the occipital pole (1 mm thickness). A high-resolution (1 mm) T1-weighted MPRAGE scan was also collected to submit to Freesurfer (Dale et al., 1999; Fischl et al., 1999, 2002) for averaging and segmentation.

As in Peterson et al. (2012), an ROI analysis was employed to interrogate visual cortex using the retinotopy data. Each participant’s V1, V2 and V4 were delineated for each visual field and projected into their individual Freesurfer space^3^. In order to identify regions in each of these visual areas that were specifically sensitive to the white figures in our displays, we used activation elicited by the *Control Novel* condition in the contralateral visual field to define our ROIs (as in Peterson et al., 2012). To do so, parameter estimates^4^ from the *Control Novel* conditions for each visual field were projected onto the appropriate hemisphere in Freesurfer space. Suprathreshold (*Z* > 1.96) voxels that were contiguous with the peak activation in each region of visual cortex were assigned to that region’s ROI (see Supplementary Figure S2 for example visual cortex ROIs in one participant). The masks for each participant were then projected back into their individual high-resolution anatomical space in FSL. Featquery (Smith et al., 2004) was then used to extract the mean parameter estimates for the *Familiar* and the *Part-Rearranged Novel* conditions from the ROIs in each region of visual cortex. Importantly, this analysis of the visual cortex simply subjects condition-related signal change to a mixed-mode repeated measures analysis of variance (ANOVA; it is not a cluster-based analysis).

#### Functional Connectivity Analysis Procedures

In order to investigate age-related changes in functional connectivity between the PRC and the visual cortex in the LH (where preliminary evidence of top-down influences has been previously observed, Peterson et al., 2012), a psychophysiological interaction (PPI) analysis was conducted comparing connectivity in young vs. older participants. A PPI analysis measures the covariance in BOLD activity between a pre-specified “seed” region (in our case, the PRC) and other regions (in our case, the visual cortex), thereby testing for relationships beyond those assessed by a typical GLM analysis. According to this analysis, the higher the covariance with the activity of the seed region during the task, the stronger the functional connectivity (Friston et al., 1993, 1997).

In order to reduce the possibility of biasing our PPI results in favor of one age group or the other, the seed region used was a cluster of voxels (found using easythresh, FEAT 5.98) in the LH PRC activated for *both* young and older adults, specifically in the direction of *Familiar > Control Novel > Part-Rearranged Novel*. Because there were no significant clusters of voxels activated for the linear trend in the PRC of older participants (see “Perirhinal Cortex” Section), we searched for a non-significant cluster by eliminating the *p*-value threshold in easythresh and simply locating a cluster activated equally for young and older participants (the resulting cluster size = 4 voxels, cluster *p* > 0.20)^5^.

The* Familiar > Control Novel > Part-Rearranged Novel* linear pattern of activation has been observed previously in both the LH PRC and LH visual cortex in young adults (Peterson et al., 2012) and was expected in the young adults in the present study as well; thus, we have reason to believe that these two regions are functionally connected. Given that these similar patterns were found when stimuli were in the RVF, we used the time course of RVF stimulus presentation as the psychological regressor in the GLM. For each participant, BOLD response time-series values were extracted from the LH PRC seed region and used as the physiological regressor. From these two regressors, the generalized PPI (or PPI) regressor was created; this was our measure of interest. Mean PPI parameter estimates from this generalized interaction regressor were extracted from LH V1, V2 and V4 for young and older participants in order to assess the connectivity between the PRC and the visual cortex and how it changes with age.

## Results

### Behavioral Results

Performance on the real-world/novel task, as measured by accuracy and reaction times (RTs), is shown in Table 1. Overall, participants were accurate on 68% of trials, significantly better than chance, *p* < 0.001. We subjected accuracy data to repeated measures ANOVA using the factors of age group (young, old), visual field (LVF, RVF), and condition (*Familiar, Control Novel, Part-Rearranged Novel*). A significant main effect of condition was found (*F*_(34, 2)_ = 18.00, *p* < 0.001, $\eta_{\text{p}}^{2}$ = 0.51) with participants less accurate for “real-world” than “novel” judgments. Follow-up paired-samples *t*-tests revealed that for both RVF and LVF presentation, participants were significantly less accurate in judging the *Familiar* displays than both types of novel displays [RVF: *t*_(18)_ = 4.53, *p* < 0.001, *d* = 1.98 for *Control Novel*; *t*_(18)_ = 3.99, *p* < 0.01, *d* = 1.83 for *Part-Rearranged Novel*; LVF: *t*_(18)_ = 4.84, *p* < 0.001, *d* = 2.05 for *Control Novel*; *t*_(18)_ = 3.83, *p* < 0.01, *d* = 1.71 for *Part-Rearranged Novel*]. The reduced accuracy for “real-world” silhouettes may have been due to the fact that it is difficult to recognize familiar objects in silhouette (De Winter and Wagemans, 2004; see also, Trujillo et al., 2010; Sanguinetti et al., 2014; Sanguinetti and Peterson, 2016). This difficulty is exacerbated because our stimuli depicted only portions of familiar objects and were presented in the periphery. The reduced accuracy for *Familiar* *Configurations* may also be due to response bias; 66% of the trials were novel, thus biasing participants to respond “novel” more often than “real-world.”^6^ No other main effects or interactions in the accuracy analysis approached conventional levels of significance (*p*s > 0.10).

We subjected the RTs from correct trials to a repeated-measures ANOVA using the factors of age group (young, old), visual field (LVF, RVF) and condition (*Familiar, Control Novel* and *Part-Rearranged Novel*). A marginal main effect of visual field was observed, *F*_(17,1)_ = 3.92, *p* = 0.063; participants were marginally faster to make accurate real-world/novel response when stimuli were in the LVF vs. RVF. No other main effects or interactions approached significance (*p*s > 0.12).

**Table 1:** Behavioral results.

	Familiar	Control Novel	Part-Rearranged Novel
		**Left visual field**	
**Young**	1380 (181)	1346 (144)	1373 (163)
	0.50 (0.40)	0.83 (0.04)	0.79 (0.03)
**Old**	1641 (146)	1441 (137)	1444 (139)
	0.51 (0.07)	0.78 (0.06)	0.69 (0.07)
		**Right visual field**	
**Young**	1378 (160)	1449 (151)	1423 (153)
	0.48 (0.05)	0.85 (0.03)	0.78 (0.04)
**Old**	1637 (106)	1552 (167)	1510 (174)
	0.53 (0.07)	0.73 (0.06)	0.74 (0.05)

*Mean RTs (top) and accuracy (bottom) by condition, visual field and age group. Note: Standard error of the mean is in parentheses adjacent to each mean*.

To ascertain that participants did not trade RT for accuracy to a greater extent in one age group than the other, we conducted an inverse efficiency analysis, which takes into account both RT and accuracy by dividing each participant’s mean RT by their proportion correct (Townsend and Ashby, 1983). An ANOVA on these scores revealed the same effects observed in the analysis of accuracy—that is, a main effect of condition (with worse performance for real-world vs. novel judgments) and no effects of or interactions with age (*p* > 0.30). This analysis indicates that a speed/accuracy trade-off cannot account for our behavioral results.

That there was no behavioral difference between young and older adults indicates that any neural differences between the two age groups are not confounded by accuracy or RTs (see “The Temporopolar Cortex” Section for a theoretical discussion on our lack of age-related behavioral differences).

### Medial Temporal Lobe Analysis Results

#### Perirhinal Cortex

Results of the linear trend analysis in the MTL are summarized in Table 2.

**Table 2:** Clusters showing significant linear trend in the medial temporal lobe.

ROI	Visual field	Age/contrast	Linear trend direction	Cluster *p*-value	Max *Z*	Peak coordinates (*x, y, z*)
LH PRC	RVF	Young	Familiar > Control novel > Part-rearranged novel	0.04	3.76	−40, −12, −20
		Young > old	Familiar > Control novel > Part-rearranged novel	0.02	3.38	−22, −4, −36
	LVF	Young > old	Familiar > Control novel > Part-rearranged novel	0.02	2.95	−22, −20, −18
LH TPC	RVF	Old > young	Part-rearranged novel > Control novel > Familiar	0.05	3.01	−32, 18, −34
RH TPC	LVF	Old > young	Part-rearranged novel > Control novel > Familiar	0.03	3.60	46, 10, −34
		Old	Part-rearranged novel > Control novel > Familiar	0.07	3.41	28, 14, −32

*Note: RVF, right visual field; LVF, left visual field; LH, left hemisphere; RH, right hemisphere; PRC, perirhinal cortex; TPC, temporopolar cortex*.

**Figure 4:**
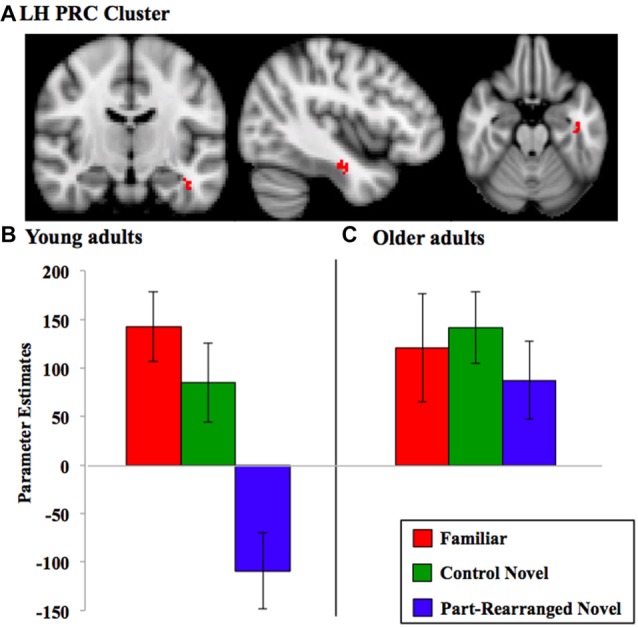
Perirhinal cortex (PRC) linear trend cluster analysis results. **(A)** PRC cluster (in red) that exhibited a significant linear pattern of activation for RVF presentation in the direction *Familiar* > *Control Novel* > *Part-Rearranged Novel* in the young participants. Parameter estimates extracted from this cluster for each condition are shown for **(B)** young and **(C)** older adults. Note that the cluster shown in **(A)** was identified via a voxelwise analysis for the group of subjects, whereas the parameter estimates were later calculated for each condition across the spatial extent of that cluster. Hence, the data in **(B)** are for illustrative purposes only; they are not an exact representation of the data submitted to the group level GLM analysis that produced the cluster. Error bars represent standard errors of the mean. LH, left hemisphere.

A mixed-design higher-level analysis was conducted to assess this linear trend within and between the two age groups (see “Medial Temporal Lobe Analysis Procedures” Section). In young participants, RVF stimulation produced a significant cluster of voxels showing a linear response trend (*Familiar > Control Novel > Part-Rearranged Novel*) in the LH PRC (max *Z* = 3.76, *p* = 0.04, cluster size = 22 voxels). This cluster is shown in Figure 4A^7^. The presence of this significant cluster indicates that for RVF stimuli, the contralateral (LH) PRC showed the highest activation to *Familiar Configurations*, less activation to *Control Novel Configurations* and the lowest activation to *Part-Rearranged Novel Configurations*. The group level statistical analysis was performed on a voxelwise basis. In Figure 4B, we show the values for each condition across the spatial extent of the cluster that was statistically significant for the entire group. The data in Figure 4B are for illustrative purposes only; they are not an exact representation of the data submitted to the group level GLM analysis (Again for illustrative purposes, individual participants’ mean data are shown in Supplementary Figure S3). No significant linear trend was observed in the young PRC for LVF presentation. These results replicate our previous research that also showed a significant linear trend in the PRC for RVF (but not LVF) presentation in young participants (Peterson et al., 2012).

In older adults, neither RVF nor LVF presentation produced a significant linear trend (*Familiar > Control Novel > Part-Rearranged Novel*) in either the left or right PRC (*p*s > 0.10); in fact, activation levels between the three conditions did not differ (see Figure 4C for RVF results).

We found significant young > old differences in the LH PRC for both RVF and LVF presentation (max *Zs* = 3.38 and 2.95, *p*s = 0.02 and 0.02, cluster size = 30 and 21 voxels, respectively); young adults showed a significantly greater linear trend (i.e., a greater difference between each of the three conditions) than the older participants.

In sum, these findings suggest that the LH PRC is sensitive to the familiarity of object configurations as well as the familiarity of their parts in young but not in older participants. For RVF presentation, then, the young-older difference is due to a significant linear trend in the young PRC. For LVF presentation, there was no significant effect in the young PRC; thus, the difference between age groups likely arose due to activation patterns trending non-significantly in opposite directions.

#### Temporopolar Cortex

A comparison between the patterns of activation in young vs. older participants in the TPC revealed significant old > young differences in both the LH TPC with RVF presentation (max *Z* = 3.01, *p* = 0.05, cluster size = 15 voxels) and the RH TPC with LVF presentation (max *Z* = 3.60, *p* = 0.03, cluster size = 26 voxels). In the RH TPC, older participants showed a marginally significant linear trend in the direction of *Part-Rearranged Novel* > *Control Novel* > *Familiar* (max *Z* = 3.41, *p* = 0.07, cluster size = 16 voxels) with LVF presentation (see Supplementary Figure S4)—the pattern Peterson et al. (2012) observed in young adults. Young participants showed no such trend. In the LH (with RVF presentation), neither young nor older adults showed a significant linear trend in either direction (*p*s > 0.10); hence the old > young difference in the LH is likely due to patterns trending in opposite directions in the two age groups, though non-significantly.

### Visual Cortex Analysis Results

A preliminary analysis of the visual cortex data revealed greater activation in LH visual cortex when the stimuli fell within the right (contralateral) visual field, and in RH visual cortex when the stimuli fell within the left (contralateral) visual field (*p*s < 0.05). These differences are expected for participants who successfully maintained fixation during stimulus presentation. This ability to maintain fixation was confirmed in the eye movement data acquired during scanning, which also showed no difference in eye movements between age groups or conditions.

**Figure 5:**
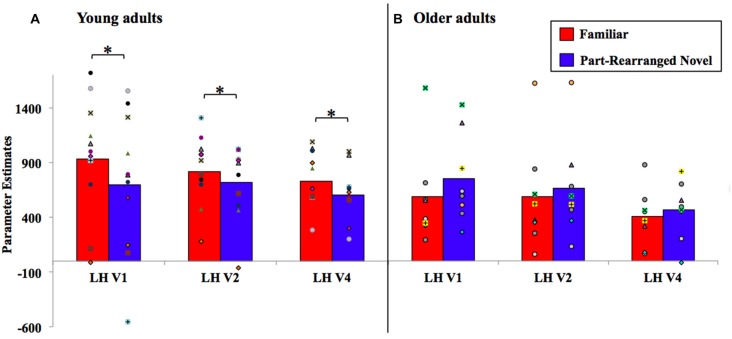
Visual cortex analysis results for **(A)** young and **(B)** older adults, with individual participants’ data superimposed. LH, left hemisphere. *P < 0.05.

Within each visual area (V1, V2 and V4 in the LH and RH), we subjected parameter estimates resulting from stimuli presented to the contralateral visual field to a repeated measures ANOVA with factors of age group (young, older) and configuration type (*Familiar, Part-Rearranged Novel*). Recall that *Control Novel Configuration* parameter estimates from each visual field were not eligible for analysis because they were used to establish our ROIs in each region of visual cortex. The results are shown in Figure 5. In the LH (with RVF presentation), a significant interaction between age and configuration type was observed in each visual area (V1, V2 and V4; *F*s_(17,1)_ = 5.14, 5.87, and 5.81, *p*s = 0.03, 0.03, and 0.02, $\eta_{\text{p}}^{2}$ *s* = 0.23, 0.26 and 0.26, respectively). One-tailed, paired-samples *t*-tests revealed, as predicted, that these interactions arose due to significantly greater activation resulting from *Familiar* than from *Part-Rearranged Novel Configurations* (the direction predicted by our prior findings) in each visual area in young participants (*t*s_(10)_ = 1.86, 2.79, 2.98, *p*s = 0.05, 0.02, 0.01, *d*s = 0.44, 0.32, 0.57 for V1, V2 and V4, respectively). We note that, as observed in Peterson et al. (2012), this pattern of activation in the visual cortex of young participants mirrors the pattern of activation observed in the PRC—that is, greater activation for *Familiar* than *Part-Rearranged Novel* *Configurations* in the LH with RVF presentation, which has been taken to suggest that the PRC modulates V2 activity. No significant differences between responses to *Familiar* vs. *Part Rearranged Novel Configurations* were present in any visual area in older participants (*p*s > 0.15). The presence of different age-dependent patterns of visual cortex responses to the same parts as a function whether they were present in a familiar vs. a novel configuration is consistent with the proposal that part familiarity responses in visual cortex are modulated by the PRC in young participants (for further discussion, see “The Perirhinal Cortex” Section). In the next section, we explore the functional connectivity between LH PRC and V2 in young and older adults.

In the RH visual cortex (with LVF presentation), no significant differences were observed between *Familiar* vs. *Part-Rearranged Novel Configurations* in either young or older participants in V1, V2, or V4 (*p*s > 0.25)^8^. This is not unexpected given the lack of an effect in the RH PRC with LVF presentation. See “General Discussion” Section for further discussion of laterality.

### Functional Connectivity Analysis Results

**Figure 6:**
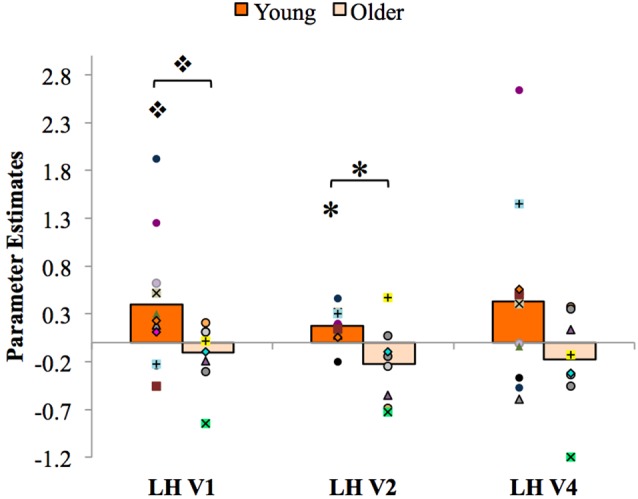
Psychophysiological interaction (PPI) analysis results, with individual participants’ data superimposed. LH, left hemisphere. *p < 0.05, *p < 0.01.

The results of the PPI analysis assessing functional connectivity between the LH PRC and the LH visual cortex and the effects of age are shown in Figure 6. Within each age group (young and older) and visual area (LH V1, V2 and V4), a one-sample *t*-test was conducted to assess the strength of the coupling with the PRC. The results showed that PPI values in young adults were significantly greater than zero in LH V2 (*t*_(10)_ = 3.65, *p* = 0.01, *d* = 1.58) and marginally greater than zero in LH V1 (*t*_(10)_ = 1.99, *p* = 0.09, *d* = 0.84). These results reveal for the first time that the LH PRC is functionally connected to the visual cortex (specifically V2) in younger adults, consistent with the hypothesized top-down modulation of V2 responses by the PRC (Barense et al., 2012; Peterson et al., 2012). PPI values in older adults were not greater than zero in any visual area (*p*s > 0.18). Thus, PRC-V2 connectivity is reduced to zero in older adults.

To further assess age-related changes in functional connectivity between the PRC and the visual cortex, independent-samples *t*-tests were conducted comparing mean PPI values in young vs. older adults in each visual area. The results showed significantly higher PPI parameter estimates in LH V2 for young than for older participants, *t*_(17)_ = 3.12, *p* = 0.01, *d* = 1.42; that is, when stimuli were presented to the RVF, the functional coupling between LH PRC and LH V2 was significantly stronger for young adults than for older adults. PPI parameter estimates in LH V1 were marginally greater for young than for older participants, (*t*_(17)_ = 1.87, *p* = 0.08, *d* = 0.91). No significant differences in PPI parameter estimates were observed within or between young and older adults in area V4 (*p*s > 0.12).

## General Discussion

The purpose of the current study was to assess whether aging compromises the sensitivity of certain MTL structures—particularly, the PRC and the TPC—to configural as well as part familiarity. Moreover, our use of a PPI analysis allowed us to investigate functional connectivity between the PRC and the visual cortex (where part familiarity is represented) in both young and older participants.

### The Perirhinal Cortex

As predicted, the PRC of older adults was significantly less sensitive than that of younger adults to the familiarity of object configurations. Consistent with our previous results, we found that the PRC of young adults was specifically sensitive to the configuration of familiar object parts. They showed a significant linear trend of activation (*Familiar* > *Control Novel* > *Part-Rearranged Novel*) in the LH PRC during RVF presentation. We note that such a pattern could only be elicited from a structure engaged in processing familiarity at both the configural and part levels; discrimination at only the configuration level would produce equivalent levels of activation for both types of novel configuration (*Control Novel* and *Part-Rearranged Novel*) while discrimination at only the part level would have produced equivalent activation for both types of configuration composed of familiar parts (*Familiar* and *Part-Rearranged Novel*). The pattern of PRC activation was significantly different in older adults, who showed no sensitivity to the configuration of familiar object parts. These results are the first to show that the sensitivity of the human PRC to object familiarity at both the part and configuration level declines with age. This age-related effect is consistent with previous research showing decline in the PRC’s object perception capabilities with age (Insel et al., 2008; Burke et al., 2010, 2011; Ryan et al., 2012). However, these prior studies have investigated age-related decline in the PRC’s involvement in configuration-level object discrimination, such as between objects with high feature ambiguity. We have added to this literature by showing age-related decline in the PRC’s sensitivity to the joint familiarity of configurations and their parts as well as to complex object configurations.

Like the PRC, older adults’ visual cortex was significantly less sensitive than younger adults’ to configural familiarity. Young adults’ LH visual cortex activity resembled that of their LH PRC—that is, higher activation for *Familiar* vs. *Part-Rearranged Novel Configurations* in LH V1, V2 and V4. Older adults, in contrast, did not show this pattern in any region of visual cortex. This likely reflected an age-related difference in top-down modulation of visual cortex. As described in models of PRC function (Bussey and Saksida, 2002; Bussey et al., 2002; Cowell et al., 2006; Murray et al., 2007), the individual features or parts of an object are represented at lower levels of the visual hierarchy (such as V2) and assembled into conjunctions of those features in the PRC. Consistent with this and as noted in previous work (Peterson et al., 2012), the RFs of V2 neurons are only large enough to encompass the parts in our displays (~1–2°). Those parts were identical in the *Familiar* and* Part-Rearranged Novel Configurations*. The difference between these two types of displays was at the configuration level; thus, that low-level visual cortex neurons responded differentially to them might suggest that they were modulated by feedback from some higher level where the RFs are large enough to encompass the entire configuration (6°)—a role for which the PRC is a likely candidate based on its whole object representations (Bussey and Saksida, 2002; see also Barense et al., 2012; Peterson et al., 2012). In the current article, we further assessed this feedback hypothesis by conducting a PPI analysis, which showed a significant difference in LH PRC and V2 functional connectivity between the two groups. Young adults showed significant connectivity between the two regions, with LH PRC activity predicting LH V2 response. This finding adds critical evidence supporting our claim that the difference in V2 activation between *Familiar* and *Part-Rearranged Novel Configurations* arises due to feedback from the PRC. In older adults, however, the functional connectivity from the PRC to V2 was not significantly different from zero. These findings are consistent with the anatomical data showing that although the PRC itself remains structurally intact with age (Insausti et al., 1998; Rapp et al., 2002), its structural connections do not. In particular, the inferior longitudinal fasciculus, which connects the temporal and occipital lobes, undergoes significant age-related deterioration (Voineskos et al., 2012). Thus, both bottom-up and top-down communication between the visual cortex and the PRC may be compromised with age, which may account both for the behavioral and functional results we describe here and those more broadly reported within the literature.

One such broader age-related functional change observed in prior studies is older adults’ reduced object perception abilities (Burke et al., 2011; Ryan et al., 2012; Scheerer and Marrone, 2014; see “Introduction” Section above). Recent data suggests that this functional deficit may in part be attributed to an age-related reduction in the inhibitory processing necessary for figure-ground segregation (specifically, inhibition of the groundside; Anderson et al., 2016; Lass et al., 2017). For instance, Anderson et al. (2016) demonstrated that compared to young adults, older adults are slower and less accurate to categorize enclosed silhouettes as “novel” when a portion of a meaningful object is suggested on its groundside. Such instances result in a high degree of cross-border competition for figural status that requires greater ground suppression in order to be resolved (Salvagio et al., 2012; Cacciamani et al., 2015; Sanguinetti et al., 2016). The reduced ability for older adults to inhibit the meaningful object in the ground to resolve that competition leads to poorer behavioral performance (Anderson et al., 2016). This inhibition involved in figure-ground segregation may be occurring in the visual cortex (Cacciamani et al., 2015; Sanguinetti et al., 2016) and may undergo age-related decline. Indeed, other researchers have shown that gamma-aminobutyric acid (GABA) receptors, which are inhibitory in nature and crucial in determining neural responses in the visual cortex (Pernberg et al., 1998), degrade with old age (Leventhal et al., 2003). An age-related decline in inhibition may help to explain the results of the current study as well, since inhibition is indeed necessary—specifically, the inhibition of familiar parts when they are present in a novel configuration (the *Part-Rearranged Novel Configurations*)—which we posit originates from higher levels and is applied to neural responses in the visual cortex.

Our findings contribute more generally to our understanding of the relationship between the PRC and visual cortex in figure-ground segregation. The functional connectivity from the PRC—as well as its age-related decline—was most evident in V2 (Figure 6); this was not unexpected. V2 neurons have RF sizes that most closely match the size of one part of our displays (~1–2°); thus, we would expect part familiarity responses to be strongest here and hence for modulation from higher areas to be strongest in young adults and most affected by aging. In their study, Peterson et al. (2012) only found their part familiarity effect in V2, giving us even more reason to expect it there. Twice as many participants were tested in the current study as were tested by Peterson et al. (2012), which allowed us to observe part familiarity effects in V4 and V1 as well as V2 (see Figure 5A), and statistically significant PRC-V2 functional connectivity as well as marginally significant PRC-V1 connectivity (see Figure 6). Therefore, the PRC may be providing feedback to multiple levels of the visual cortex. Consistent with this suggestion, research on rats has shown that multiple areas of the visual cortex—specifically, the equivalent of human V1 and V2—receive direct projections from the PRC (Miller and Vogt, 1984). The existence of these structural pathways further supports the claim that the PRC modulates low-level visual regions (Barense et al., 2012; Peterson et al., 2012), and may even underlie the functional connectivity observed between the PRC and V2 in young adults in the present study. We acknowledge that a PPI analysis is only correlative and thus does not allow us to speak to direct modulation of responses. In order to gain a more direct measure of top-down influences, a causal analysis would be needed. Additionally, although we did have more participants in this study than in Peterson et al. (2012), we were only able to analyze eight older adults (compared to 11 young adults), which may complicate our interpretation of the null results observed in that age group. However, Peterson et al. (2012) observed a significant linear trend in the PRC with only six young participants, which led us to believe that the effect is robust when it exists. Future studies are needed to investigate this.

Although age-related differences in activation patterns were observed in the LH PRC for both RVF and LVF stimuli presentation, the significant linear effect (*Familiar* > *Control Novel* > *Part-Rearranged Novel*) was only apparent in the young during RVF presentation. This effect of visual field was also observed by Peterson et al. (2012); however, in the present study, only the LH (contralateral) PRC exhibited the linear pattern with RVF presentation, whereas in Peterson et al. (2012) it was apparent bilaterally. That both studies observed significant differences in the LH PRC for RVF presentation, though, suggests that the LH might be particularly involved in processing the familiarity vs. novelty of configurations and their parts, which is why the effect has consistently been observed there. This is not to say that the RH has no role in familiarity/novelty discrimination—just that the LH might be driving the effect. Future studies could elucidate laterality effects in familiarity/novelty effects.

### The Temporopolar Cortex

In their study, Peterson et al. (2012) found that the TPC was sensitive to the familiarity of the configuration as well as of the parts of stimuli presented in the LVF (although the pattern of activation—*Part-Rearranged Novel* > *Control Novel* > *Familiar*—was the opposite of the pattern observed in the PRC). In the present study, with twice the number of participants, we found no clusters of voxels exhibiting significant differences in activation between the three conditions in the TPC in young participants. These inconsistent findings in the TPC might indicate that it is not as sensitive to configural and part familiarity as the PRC in which we have consistently observed significant differences in activation between the three conditions in young participants. An alternative explanation may lie in the different pulse sequences (and even magnets) used to acquire fMRI data in the two experiments. The previous experiment employed a spiral-in, spiral-out sequence, which produces relatively low spatial resolution but is optimal for avoiding signal drop out that typically plagues data collected from the medial and anterior temporal lobes (Both regions lie close to anatomical structures that cause inhomogeneities in the local magnetic field). The current experiment was performed using an EPI sequence, which provides higher spatial resolution and thus finer localization of activity in visual cortex, but is less robust to signal drop out from anterior ventral regions of the temporal cortex. Regardless of the reason, in the present experiment, we observed no significant effects in the TPC of young adults and only marginally significant effects in the TPC of older adults. This fact, combined with the fact that no lower-level visual cortex responses mimicked any patterns of activation observed in the TPC (in young or older adults), precluded us from conducting a functional connectivity analysis between the TPC and the visual cortex.

Our investigation of age revealed a significant difference in the pattern of activation in the TPC of young vs. older adults (bilaterally, during both LVF and RVF stimulus presentation), however, with older adults showing a significantly greater linear trend in the direction of *Part-Rearranged Novel* > *Control Novel* > *Familiar* compared to the young adults. It is interesting to consider whether this recruitment of the TPC—rather than the PRC—in responding to configural/part familiarity in older participants arises as a way to compensate for the PRC’s decline. Indeed, other research has shown that neural compensation and reorganization is a mechanism by which the brain copes with age-related decline (Cabeza et al., 2002; Reuter-Lorenz and Lustig, 2005). In the present study, the PRC was shown to represent configural/part familiarity in young but not older adults (our primary result of interest). Older adults were more likely to use the TPC to represent familiarity than young adults. Thus, the neural mechanism necessary for the behavioral assessment of familiarity still exists in our sample of older adults; it just does not include the PRC. Consistent with this suggestion, younger and older adults were equally able to identify familiar configurations as such.

Our lack of behavioral differences can be considered a strength of the present study for another reason: because we found no differences between age groups, we can be sure that age-related reductions in signal strength reflect reductions in neural activation rather than reductions in model robustness. The models we derived for individual participants included only correct trials. If the two groups had shown behavioral differences, the models generated for older adults would have included fewer events than those generated for younger adults and would therefore have been weaker (i.e., less likely to detect activity) than those generated for younger adults. Because we have the same number of events in the models generated for the two groups, however, we can be confident that our results reflect older adults’ failure to activate our ROI.

To help understand our lack of behavioral differences between age groups, we can also look to how our study differs from previous research showing age-related decrements in object perception. For instance, we presented each object individually, whereas other studies presented multiple complex objects simultaneously and asked participants to make a same/different judgment about them (Ryan et al., 2012; Scheerer and Marrone, 2014). It is possible that age-related deficits in object perception are more evident under conditions of high ambiguity, as defined by overlapping features between two simultaneously presented objects rather than conjunctions of features within one object. Moreover, the features (parts) in our stimuli may be less complex than those used in other studies, given that we used silhouettes that lack internal details. Perhaps, given this fact, the older adults did not need to recruit the PRC to perform our task, and instead could rely on other structures less sensitive to *complex* feature conjunctions, which may include the TPC. Further work is necessary to explicate the conditions under which impaired object perception arises in older adults, as well as the role of the TPC in object and part familiarity.

## Conclusions

Using fMRI, this study uncovered the novel finding that the PRC undergoes age-related decline in its sensitivity to the familiarity of not only complex object configurations (as others have found), but also of the parts that comprise those configurations. This was particularly the case in the LH during RVF stimulus presentation—a laterality effect similar to that observed in a previous study using the same design (Peterson et al., 2012). Like Peterson et al. (2012) we found that activation in the LH visual cortex mimicked that of the LH PRC in young participants (during RVF presentation), consistent with the hypothesis that the PRC modulates V2 responses to familiar parts as a function of whether are presented in a familiar or a novel configuration. A PPI analysis provided evidence supporting this PRC-V2 modulation hypothesis in young participants as well as an age-related decline in PRC-to-V2 functional connections. The TPC—a structure that previously showed sensitivity to configural/part familiarity in the young (Peterson et al., 2012) albeit via responses in the opposite direction from the PRC—showed that pattern in older but not younger adults in the present study. Together, these results substantially extend the field’s understanding of the age-related changes that occur in the MTL during the assessment of an object’s familiarity.

## Ethics Statement

This study was carried out in accordance with the recommendations of the Institutional Review Board at the University of Arizona. All subjects gave written informed consent in accordance with the Declaration of Helsinki. The protocol was approved by the Institutional Review Board at the University of Arizona.

## Author Contributions

PES and MAP initiated and conceived the study. LC, EW and PES recruited and ran the participants and analyzed the data. All authors participated in discussion and writing of the final manuscript.

## Conflict of Interest Statement

The authors declare that the research was conducted in the absence of any commercial or financial relationships that could be construed as a potential conflict of interest.

## Footnotes

^1^The fact that we had to eliminate participants from the analysis due to poor performance was not unexpected. Even with the pre-scanning training session, the task was very difficult given the peripheral location of the stimuli.

^2^Pilot testing with these stimuli indicated that participants did indeed perceive the *Familiar Configurations* as familiar and the *Control Novel Configurations* as novel. Importantly, the *Part-Rearranged Novel Configurations* were also perceived as novel; participants did not consciously recognize the familiar parts (but we expect that the brain still processes them as such).

^3^The delineation of visual area VP was difficult to determine because the representations of its borders were often overlapping—an issue that others have encountered previously (Shipp et al., 1995). Thus, VP was not included in any analyses.

^4^We note that percent signal change (rather than parameter estimates) is often used in these types of analyses. Percent signal change typically involves scaling the parameter estimates. Although the scaling factor is usually the inverse of the mean signal, other factors may be used. As such, analyses of percent signal change are more vulnerable to methodologically introduced variability than are analysis of raw parameter estimates. Therefore it is our standard practice to analyze data from raw parameter estimates (Scalf and Beck, 2010; Scalf et al., 2011; Peterson et al., 2012; Cacciamani et al., 2015; Simon et al., 2017).

^5^We note that in a previous version of this analysis, we used the region in which young adults showed a significant linear trend as the seed ROI and found the same results that we show using the less biased seed region we report here.

^6^Recall that for the fMRI analysis, the number of correct trials in each condition was equated (see “Data Acquisition and Analysis” Section).

^7^The PRC cluster shown in Figure 4A was produced from a group analysis and projected onto an average brain. Thus, there were multiple transformations that occurred when moving from an individual to a group analysis, which may have slightly shifted our ROIs shown on the average brain. This cluster is located deep in the sulcus on the border of the gray and white matter, and importantly, it still falls within the larger PRC ROI probability map delineated by Devlin and Price (2007) (see Supplementary Figure S1A).

^8^In Peterson et al. (2012) young participants, RH V2 mirrored the pattern of activation observed in the RH TPC. Here, we observed an effect in the RH TPC in older (but not younger) adults; however, their RH visual cortex did not mirror that activation pattern, which may be suggestive of reduced modulation of V2 by higher-level regions in older adults.
