# Supplementary material for: Age-Related Changes in Perirhinal Cortex Sensitivity to Configuration and Part Familiarity and Connectivity to Visual Cortex
Source: Front Aging Neurosci. 2017 Sep 15;9:291. doi: 10.3389/fnagi.2017.00291 (PMC5605556; doi:10.3389/fnagi.2017.00291)
Supplement: Supplementary file 1 [file Data_Sheet_1.docx]

Supplementary Material

Age-related changes in perirhinal cortex sensitivity to configuration and part familiarity and connectivity to visual cortex

**Laura Cacciamani*, Erica Wager, Mary A. Peterson, and Paige E. Scalf**

*** Correspondence:** Corresponding Author: Lcacciamani@gmail.com

# Supplementary Figures


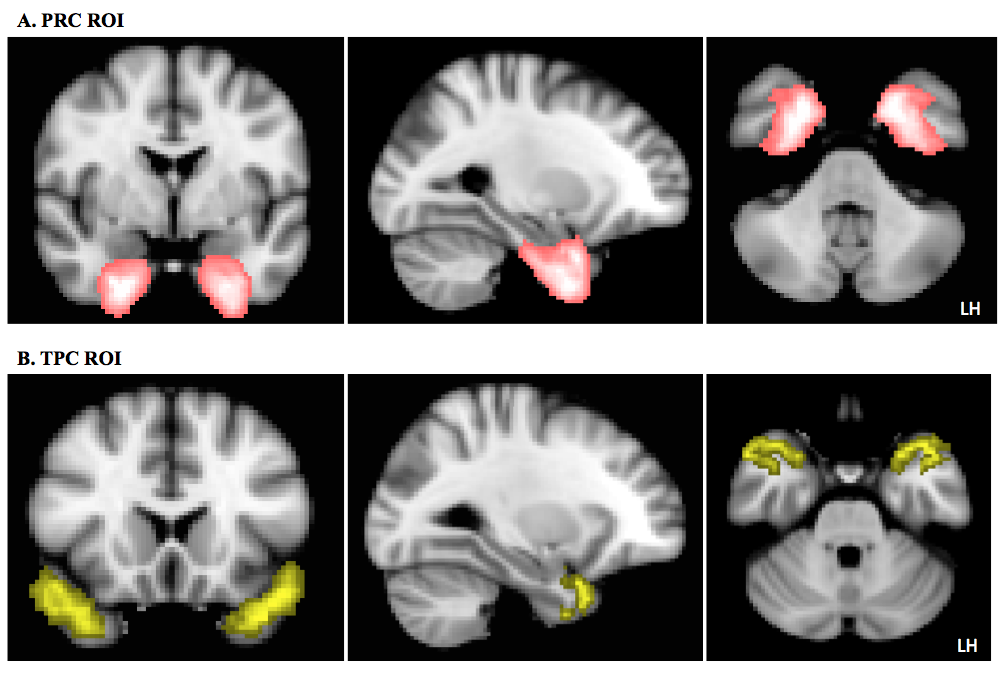


# Supplementary Figure 1. Medial temporal lobe regions of interest (ROIs) used in the current study. (A) Perirhinal cortex (PRC) ROI, shown in pink. (B) Temporopolar cortex (TPC) ROI, shown in yellow. LH = left hemisphere.


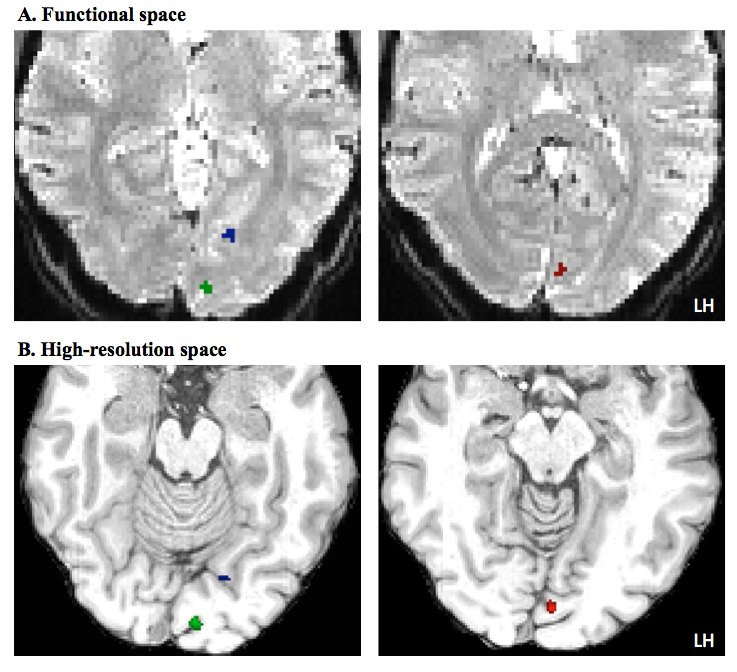


# Supplementary Figure 2. Visual cortex regions of interest (ROIs) in the LH of one representative participant, shown (A) in functional space, where the ROIs were originally created, and (B) in high-resolution anatomical space, where the ROIs were ultimately projected. ROIs were based on peak activation in response to the *Control Novel* silhouettes (see text) and were primarily located in the grey matter. V1 is shown in green, V2 in red, and V4 in blue. LH = left hemisphere.


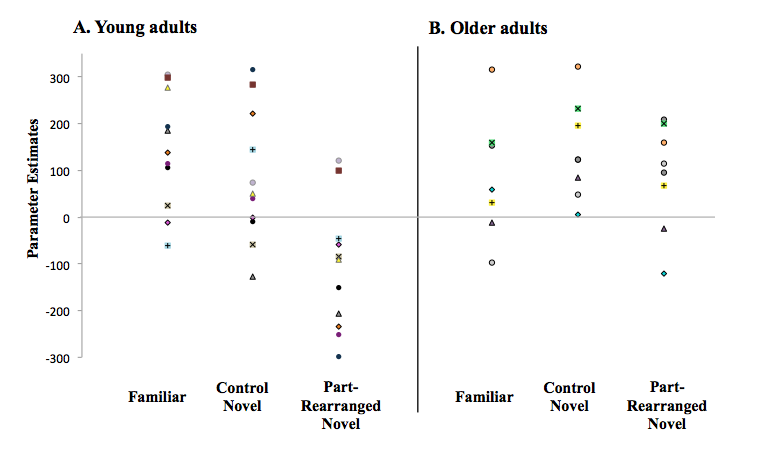


**Supplementary Figure 3**. Parameter estimates extracted from the perirhinal cortex (PRC) cluster (shown in Figure 4A) for individual (A) young and (B) older adults. Each subject is assigned a different color. These results are for illustrative purposes only because the group level statistical analysis was performed on a voxelwise basis, whereas these data are the values for each condition across the spatial extent of the cluster that was statistically significant for the entire group.

#
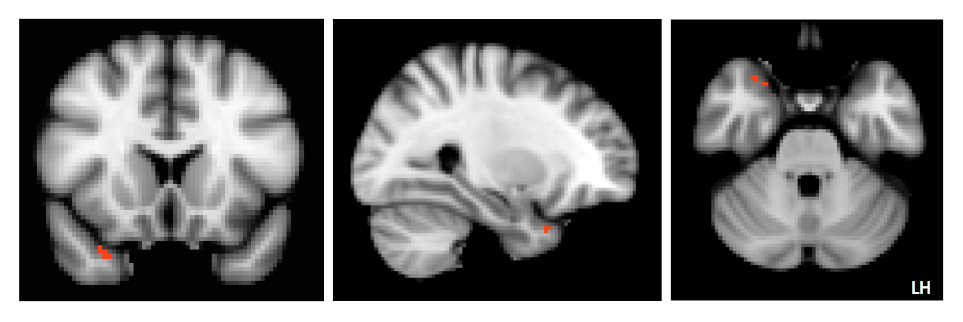


# Supplementary Figure 4. Temporopolar cortex (TPC) cluster (shown in red) that exhibited a marginally significant linear pattern of activation in the right hemisphere for LVF presentation in the direction *Part-Rearranged Novel > Control Novel* > *Familiar* for older participants. LH = left hemisphere. LVF = left visual field.
